# MRI Features of Hepatic Sarcomatoid Carcinoma Different From Hepatocellular Carcinoma and Intrahepatic Cholangiocarcinoma

**DOI:** 10.3389/fonc.2021.611738

**Published:** 2021-06-17

**Authors:** Hongbin Zhang, Siyuan Chai, Lintao Chen, Yubizhuo Wang, Yongna Cheng, Quan Fang, Guosen Wu, Xiangming Wang, Wenjie Liang, Wenbo Xiao

**Affiliations:** ^1^ Department of Radiology, Yiwu Central Hospital, Yiwu, China; ^2^ Department of Hepatobiliary and Pancreatic Surgery, The First Affiliated Hospital, Zhejiang University School of Medicine, Hangzhou, China; ^3^ Department of Radiology, The First Affiliated Hospital, Zhejiang University School of Medicine, Hangzhou, China

**Keywords:** hepatic sarcomatoid carcinoma, radiology, magnetic resonance imaging, intrahepatic cholangiocarcinoma, hepatocellular carcinoma

## Abstract

**Introduction:**

Hepatic sarcomatoid carcinoma (HSC) is a rare type of liver cancer with a high malignant grade and poor prognosis. This study compared the clinical characteristics and magnetic resonance imaging (MRI) features of HSCs with those of hepatocellular carcinoma (HCC) and intrahepatic cholangiocarcinoma (ICC), aiming to identify valuable features for HSC diagnosis.

**Methods:**

In total, 17 pathologically confirmed HSC cases, 50 HCC cases and 50 common ICC cases were enrolled from two hospitals. The clinical characteristics and MRI features of all cases were summarized and statistically analyzed.

**Results:**

On the one hand, the incidence rates of elevated carbohydrate antigen (CA) 19-9 and elevated carcinoembryonic antigen (CEA) were significantly higher in the HSC cases than in the HCC cases (29.4% *vs.* 0%; 17.6% *vs.* 0%). The HSC enhancement patterns, primarily including progressive enhancement, were also significantly different from HCC cases. The incidence rates of heterogeneous signals on T2-weighted imaging and during the arterial phase were significantly higher in the HSC cases than in the HCC cases (94.1% *vs.* 66.0%; 100.0% *vs.* 72.0%). The diameter of HSCs was significantly larger than that in the HCC cases (6.12 cm *vs.* 4.21 cm), and the incidence rates of adjacent cholangiectasis, intrahepatic metastasis and lymph node enlargement were considerably higher in the HSC cases than in the HCC cases (52.9% *vs.* 6.0%; 47.1% *vs.* 12.0%; 41.2% *vs.* 2.0%). On the other hand, the incidence rate of elevated CA199 was significantly lower in the HSC cases than in the ICC cases (29.4% *vs.* 60.0%). The incidence rates of intratumoral necrosis and pseudocapsules were significantly higher in the HSC cases than in the HCC cases (35.3% *vs.* 8.0%; 47.1% *vs.* 12.0%). However, the incidence rates of target signs were significantly lower in the HSC cases than in the HCC cases (11.8% *vs.* 42.0%). In addition, there was no significant difference in the enhancement patterns between HSC cases and ICC cases.

**Conclusions:**

HSCs were frequently seen in elderly men with clinical symptoms and elevated CA199 levels. The MRI features, including large size, obvious heterogeneity, hemorrhage, progressive enhancement, pseudocapsule and lymph node enlargement, contributed to the diagnosis of HSC.

## Introduction

Hepatic sarcomatoid carcinoma (HSC) is a rare malignant tumor that accounts for less than 4% of liver cancers (1). The pathological features of HSCs are dominated by the proliferation of spindle cells and polymorphic cells, manifesting alternate permutation with acini (2). There are two opinions about the pathogenesis of HSC, including the sarcomatoid transformation of hepatocellular carcinoma (HCC), which is more widely accepted, and the combination of liver cancer with sarcoma (3). The cause of HSCs remains unclear and is related to a small portion being resistant to anti-HCC treatment (3). The onset age of patients with HSC is approximately 60 years old (4–6). HSCs are frequently seen in men, in whom the morbidity rate is 3- to 4-fold that in women (4–6). The clinical manifestations of HSC patients are dominated by abdominal pain, followed by fatigue, fever and jaundice (7). HSCs are poorly differentiated, and half of cases are pathological grades III and IV (4, 6). More than half of HSC cases are unresectable because of peripheral invasion and extensive extrahepatic metastasis (6). In addition, short-term tumor recurrence is common in patients receiving radical surgery, with an average overall survival of only 8 months (4). Therefore, clinicians should pay attention to this type of liver cancer. It is important to analyze the imaging features of HSCs for the formulation of individualized therapeutic strategies.

In the past, a few studies focused on the imaging features of HSCs (8–10). As reported, 24 HSC cases showed peripheral enhancement and poor blood supply on contrast-enhanced computed tomography (CT)/magnetic resonance imaging (MRI) (9). Another 10 HSC cases manifested with peripheral enhancement and progressive enhancement (10). In a recent study, approximately 3 of 4 cases were classified as malignant liver tumors according to the liver imaging report and data system (LI-RADS), and the remaining 1 of 4 cases showed the features of HCC (8). These research findings suggested that the imaging results of HSCs are complicated and diverse. Thus, imaging data should be added to deepen understanding of HSCs.

In our study, the clinical characteristics and MRI features of 17 HSC cases were summarized and compared with 50 HCC and 50 intrahepatic cholangiocarcinoma (ICC) cases to determine the valuable diagnostic features of HSC.

## Materials and Methods

### Patients

This retrospective study was approved by the Ethics Committees of the First Affiliated Hospital, Zhejiang University School of Medicine, and Yiwu Central Hospital. Informed consent was waived due to the retrospective nature of this study. All of the case data were collected from the two hospitals. Patients diagnosed with “HSC” and its old item were searched in the clinical electronic medical record system from January 2010 to December 2019. The inclusion criteria for HSCs were as follows: (I) pathological confirmation of primary cases based on tissue specimens; (II) reception of conventional liver MRI and three-phase contrast-enhanced scan before surgery; and (III) complete pathological and MRI data. The exclusion criteria for HSCs were as follows: (I) lack of pathological diagnosis results; (II) reception of antitumor treatment before liver MRI scan; and (III) recurrent cases or metastatic cases. In addition, certain numbers of HCC and ICC cases were enrolled as control groups according to the inclusion and exclusion criteria of HSCs. The diagnosis of all of the cases in the three groups was confirmed by two senior pathologists.

This study finally enrolled 17 HSC patients and excluded 2 cases because of a lack of MRI images. Additionally, 50 HCC cases and 50 ICC cases were enrolled as control groups. The clinical information of patients was collected, including age, sex, symptoms, liver function, clinical stage, concurrent diseases, addiction, and serum tumor markers. The clinical data from patients in different groups were sorted and analyzed by one junior surgeon and one senior statistician.

### MRI Scan Parameters

All of the HSC and control cases underwent conventional liver MRI, contrast-enhanced MRI, and diffusion weighted imaging (DWI) sequence scans. MRI scans were performed using a GE Signa HD_XT_ 3.0-T MR system in the First Affiliated Hospital, Zhejiang University School of Medicine, and a GE Signa HDxt 1.5-T MR system (both GE Medical Systems, Milwaukee, WI, USA) in Yiwu Central Hospital. The main MRI scanning parameters in the two hospitals are summarized in **Table 1**. For contrast-enhanced scans, Gd-DTPA (0.1 mmol/kg) was administered through the elbow vein using a high-pressure injector (2.5 ml/s). The arterial phase, portal phase and hepatic vein phase were scanned at delayed times of 14-15, 45-55 and 180 s, respectively.

**Table 1:** MRI scanning parameters in the two hospitals.

	Hospital I	Hospital II	Hospital I	Hospital II	Hospital I	Hospital II	Hospital I	Hospital II
**Imaging**	T1-weighted imaging		T2-weighted imaging		Diffusion-weighted imaging		Lipid-suppression imaging	
**Breath**	Breath-hold		Respiratory gating		Respiratory gating		Breath-hold	
**Position**	Axial		Axial		Axial		Axial	
**Sequence**	Liver acceleration volume acquisition		Fast spin-echo		Echo planar imaging		Liver acceleration volume acquisition-Flex/dual-echo (in and opposed-phase)	
**TR** (ms)	3.3	6.1	Respiratory rate related individual time		Respiratory rate related individual time		4.1/4.1	200
**TE** (ms)	1.5	3.1	85	85	80	72	1.1/2.3	2.1/4.3
**Flip angle** (◦)	10/15	15	/	/	/	/	10	80
**Field of view** (cm)	38 × 30.4	38×38	38×28.5	38×38	38×28.5	38×38	38×30.4	38×38

TR, repetition time; TE, echo time.

Hospital I: The First Affiliated Hospital, Zhejiang University School of Medicine.

Hospital II: Yiwu Central Hospital.

### MRI Features

The MRI images for HSC cases and control cases were evaluated independently by two experienced radiologists who were blinded to the pathological results. The evaluation items included tumor number (single or multiple), tumor site (left lobe, right lobe or caudate lobe), maximum cross-section diameter, morphology (regular or irregular), boundary (well-defined or ill-defined), and tumor signal (compared to peripheral normal liver parenchyma). On T1WI and T2WI, the signals were recorded as hypointense, isointense, slightly hyperintense, or hyperintense, while T2WI signal consistency was evaluated as heterogeneous or nonheterogeneous. On the DWI sequence (b = 800/1000 s/mm²), the lesion signal intensity was recorded as low, equal, slightly high or high. In addition, the evaluation items also included target signs, intratumoral fat, hemorrhage, necrosis, and adjacent cholangiectasis.

Tumor enhancement on arterial phase MRI scans was classified as overall, partial, peripheral, equal or low. Other MRI features were evaluated, including tumor enhancement consistency (heterogeneous or nonheterogeneous), abnormal perfusion, peritumoral wash-in and wash-out, pseudocapsule, hepatic capsular retraction (HCR), vein tumor thrombi, mosaic sign, intrahepatic metastasis and lymph node enlargement. The enhancement patterns included wash-in and wash-out, persistently high enhancement (defined as high enhancement of partial or whole tumors in three enhanced phases), peripheral enhancement, progressive enhancement (defined as an enhanced range expanding with time), fading, and poor blood supply (defined as low enhancement during the three phases). Imaging of liver tumors was evaluated by referring to the definitions and annotations in LI-RADS (9). Two senior radiologists finally provided consistent imaging evaluation results.

### Data Statistics

SPSS software, version 24 (IBM, Chicago, IL, USA), for the Mann-Whitney U test, chi-square test and Fisher’s exact test, was used to evaluate the differences in features. Continuous variables, including age and maximum cross-section diameter, were statistically analyzed by the bilateral Mann-Whitney U test. Categorical variables (such as clinical characteristics and imaging features) were analyzed statistically by bilateral chi-square tests. Variables with a frequency of 0 and groups with a sample size < 40 were investigated by Fisher’s exact test. *p* < 0.05 and *p* < 0.01 indicate significant differences between the two groups.

## Results

### Clinical Characteristics

The 17 HSC cases consisted of 14 men and 3 women. The onset age was 28-76 years old, with an average of 59.2 ± 13.7 years old. Fourteen patients had clinical symptoms, while three were asymptomatic. Nine patients had abdominal pain and abdominal distension, two had abdominal distention combined with weakness, one had yellow urine and poor appetite, one had nausea, and one had fever. Concurrent diseases included hepatitis B virus (HBV) infection in 10 cases, hepatitis C virus (HCV) infection in 1 case, liver cirrhosis in 8 cases, chronic liver disease (CLD) in 10 cases, cholelithiasis in 5 cases, cholangiolithiasis in 1 case, hypertension in 4 cases and diabetes in 3 cases. Addictions included smoking in 6 cases and alcohol abuse in 8 cases. Plasma tumor markers included elevated carbohydrate antigen (CA) 12-5 levels in seven cases, elevated CA 19-9 levels in five cases, elevated carcinoembryonic antigen (CEA) levels in three cases and elevated α-fetoprotein (AFP) levels in two cases.

There were 50 HCC cases and 50 ICC cases as the control group. The clinical characteristics of the three groups of patients are summarized in **Table 2**.

**Table 2:** Clinical features and statistical results of HSC group compared to the HCC and ICC groups.

	HSC (n = 17)	HCC (n = 50)	*HSC *vs.* HCC p*	ICC (n = 50)	*HSC vs. ICC p*
**Sex**			0.401		0.052
Male	14 (82.4%)	45 (90.0%)		28 (56.0%)	
Female	3 (17.6%)	5 (10.0%)		22 (44.0%)	
**Age** (years)	59.2 (± 13.7)	56.5 (± 11.2)	0.286	60.7 (± 10.4)	0.965
**Symptoms**			<0.001		0.019
Symptomatic	14 (82.4%)	15 (30.0%)		25 (50.0%)	
Asymptomatic	3 (17.6%)	35 (70.0%)		25 (50.0%)	
**Hepatitis C infection**			1.000		0.254
Yes	1 (5.9%)	2 (4.0%)		0	
No	16 (94.1%)	48 (96.0%)		50 (100%)	
**Hepatitis B infection**			0.001		0.441
Yes	10 (58.8%)	46 (92.0%)		24 (48.0%)	
No	7 (41.2%)	4 (8.0%)		26 (52.0%)	
**Chronic liver disease**			0.012		0.181
Yes	12 (70.6%)	48 (96.0%)		26 (52.0%)	
No	5 (29.4%)	2 (4.0%)		24 (48.0%)	
**Liver cirrhosis**			0.523		0.048
Yes	8 (47.1%)	28 (56.0%)		11 (22.0%)	
No	9 (52.9%)	22 (44.0%)		39 (78.0%)	
**Cholelithiasis**			0.152		0.025
Yes	5 (29.4%)	7 (14.0%)		4 (8.0%)	
No	12 (71.6%)	43 (86.0%)		46 (92.0%)	
**Cholangiolithiasis**			0.254		0.254
Yes	1 (5.9%)	0		0	
No	16 (94.1%)	50 (100%)		50 (100%)	
**Liver function**			0.658		0.594
A	10	26		33	
B	7	24		17	
**Clinical stage**			<0.001		0.175
I/II	2	32		14	
III/IV	15	18		36	
**Hypertension**			0.757		0.757
Yes	4 (23.5%)	10 (20.0%)		10 (20.0%)	
No	13 (76.4%)	40 (80.0%)		40 (80.0%)	
**Diabetes**			0.555		0.832
Yes	3 (17.6%)	6 (12.0%)		10 (20.0%)	
No	14 (82.4%)	44 (88.0%)		40 (80.0%)	
**Smoking**			0.442		0.570
Yes	6 (54.5%)	23 (46.0%)		14 (28.0%)	
No	11 (64.7%)	27 (54.0%)		36 (72.0%)	
**Alcohol abuse**			0.263		0.106
Yes	8 (47.0%)	16 (32.0%)		13 (26.0%)	
No	9 (52.9%)	34 (68.0%)		37 (74.0%)	
**Elevated CA125 level**			<0.001		0.594
Yes	7 (41.2%)	0		17 (34.0%)	
No	10 (58.8%)	50 (100%)		33 (66.0%)	
**Elevated CA199 level**			0.001		0.029
Yes	5 (29.4%)	0		30 (60.0%)	
No	12 (70.6%)	50 (100%)		20 (40.0%)	
**Elevated CEA level**			0.014		0.832
Yes	3 (17.6%)	0		10 (20.0%)	
No	14 (82.4%)	50 (100%)		40 (80.0%)	
**Elevated AFP level**			0.008		0.062
Yes	2 (11.8%)	24 (48.0%)		0	
No	15 (88.2%)	26 (52.0%)		50 (100%)	

HSC, Hepatic sarcomatoid carcinoma; ICC, intrahepatic cholangiocarcinoma; HCC, hepatocellular carcinoma; CA125, carbohydrate antigen 12-5; CA199, carbohydrate antigen 19-9; CEA, carcinoembryonic antigen; AFP, alpha fetoprotein.

### Comparison of Clinical Characteristics

There were significant differences in plasma tumor markers (CA125, CA199 and AFP), symptoms, clinical stage and hepatitis B infection between the HSC group and the HCC group (*p* < 0.01). There were significant differences in tumor markers (CA199), clinical symptoms, liver cirrhosis and cholelithiasis between the HSC group and the ICC group (*p* < 0.05). There were no statistically significant differences in other clinical features between the HSC and control groups.

### Conventional MRI Features

Among the 17 HSC cases, 10 had a single lesion, while 7 had multiple lesions. The maximum cross-sectional diameter of the tumors was approximately 2.0-11.0 cm, with an average of 6.1 ± 3.2 cm. The tumor site was the right lobe in 13 cases and the left lobe in 4 cases. The shape of the tumor was regular in 9 cases and irregular in 8 cases. In addition, the tumor boundary was well defined in 14 cases and ill defined in 3 cases. The 17 HSC cases showed hypointensity on the T1WI sequence and slight hyperintensity on the T2WI sequence (nonheterogeneous in 1 case and heterogeneous in 16 cases). On the DWI sequence, all 17 cases showed high signals. Additionally, nine cases had concurrent adjacent cholangiectasis, nine showed mosaic signs, eight had intrahepatic metastasis, seven had lymph node enlargement, six had intratumoral hemorrhage, six had intratumoral necrosis, and two had HCR.

The conventional MRI features in the HSC, HCC and ICC groups are summarized in **Table 3**.

**Table 3:** MRI features and statistical results of HSC group compared to the HCC and ICC groups.

	HSC (n = 17)	HCC (n = 50)	*HSC *vs.* HCC p*	ICC (n = 50)	*HSC vs. ICC p*
**Number**			0.175		0.816
Single	10 (58.8%)	38 (76.0%)		31 (74.0%)	
Multiple	7 (41.2%)	12 (24.0%)		19 (26.0%)	
**Tumor site**			0.312		0.075
Left lobe	4 (23.5%)	22 (44.0%)		26 (52.0%)	
Right lobe	13 (76.5%)	27 (54.0%)		23 (46.0%)	
Caudate lobe	0	1 (2.0%)		1 (2.0%)	
**Maximum diameter (cm)**	6.12 ± 3.18	4.21 ± 2.38	0.021	5.12 ± 2.20	0.327
**Morphology**			0.419		0.219
Regular	9 (52.9%)	32 (64.0%)		18 (36.0%)	
Irregular	8 (47.1%)	18 (36.0%)		32 (64.0%)	
**Tumor boundary**			0.188		0.123
Well-defined	14 (82.4%)	48 (96.0%)		31 (62.0%)	
Ill-defined	3 (17.6%)	2 (4.0%)		19 (38.0%)	
**T1-weighted imaging**			0.616		–
Isointensity	0	2 (4.0%)		0	
Hypointense	17 (100%)	48 (96.0%)		50 (100%)	
**T2-weighted imaging**			0.024		0.258
Slightly hyperintense	17 (100%)	50 (100%)		50 (100%)	
Nonheterogeneous	1 (5.9%)	17 (34.0%)		11 (22.0%)	
Heterogeneous	16 (94.1%)	33 (66.0%)		39 (78.0%)	
**Diffusion weighted imaging**			0.055		0.103
High signal	17 (100%)	40 (80.0%)		42 (84.0%)	
Slightly high signal	0	10 (20.0%)		8 (16.0%)	
**Target sign**	2 (11.8%)	3 (6.0%)	0.805	21 (42.0%)	0.023
**Intratumoral fat**	0	4 (8%)	0.348	0	–
**Intratumoral hemorrhage**	6 (35.3%)	11 (22.0%)	0.277	4 (8.0%)	0.006
**Intratumoral necrosis**	6 (35.3%)	8 (16.0%)	0.091	10 (20.%)	0.201
**Adjacent cholangiectasis**	9 (52.9%)	3 (6.0%)	<0.001	37 (74.0%)	0.106
**Arterial phase**			<0.001		0.738
Integral enhancement	0	13 (26.0%)		4 (8.0%)	
Partial enhancement	10 (58.8%)	35 (70.0%)		22 (44.0%)	
Peripheral enhancement	5 (29.4%)	0		16 (32.0%)	
Low enhancement	2 (11.8%)	2 (4.0%)		8 (16.0%)	
**Enhancement consistency**			0.014		0.565
Nonheterogeneous	0	14 (28.0%)		3 (6.0%)	
Heterogeneous	17 (100%)	36 (72.0%)		47 (94.0%)	
**Abnormal perfusion**	12 (70.6%)	4 (8.0%)	<0.001	32 (64.0%)	0.621
**Peripheral wash-in wash-out**	0	0	–	2 (4.0%)	0.616
**Pseudocapsule**	8 (47.1%)	42 (84.0%)	0.002	6 (12.0%)	0.002
**Hepatic capsular retraction**	2 (11.8%)	2 (4.0%)	0.565	9 (18.0%)	0.825
**Vein tumor thrombi**	5 (29.4%)	6 (12.0%)	0.195	13 (26.0%)	1.000
**Enhancement pattern**			<0.001		0.329
Wash-in and wash-out	0	47 (94.0%)		4 (8.0%)	
Persistently high enhancement	5 (29.4%)	2 (4.0%)		11 (22.0%)	
Peripheral enhancement	0	0		1 (2.0%)	
Progressive enhancement	10 (58.8%)	0		33 (66.0%)	
Fading	0	1 (2.0%)		0	
Poor blood supply	2 (11.8%)	0		1 (2.0%)	
**Mosaic sign**	9 (52.9%)	31 (62.0%)	0.511	14 (28.0%)	0.061
**Intrahepatic metastasis**	8 (47.1%)	6 (12.0%)	0.002	15 (30.0%)	0.201
**Lymph node enlargement**	7 (41.2%)	1 (2.0%)	<0.001	29 (58.0%)	0.229

HSC, Hepatic sarcomatoid carcinoma; ICC, intrahepatic cholangiocarcinoma; HCC, hepatocellular carcinoma.

### Contrast-Enhanced MRI Features

**Figure 1:**
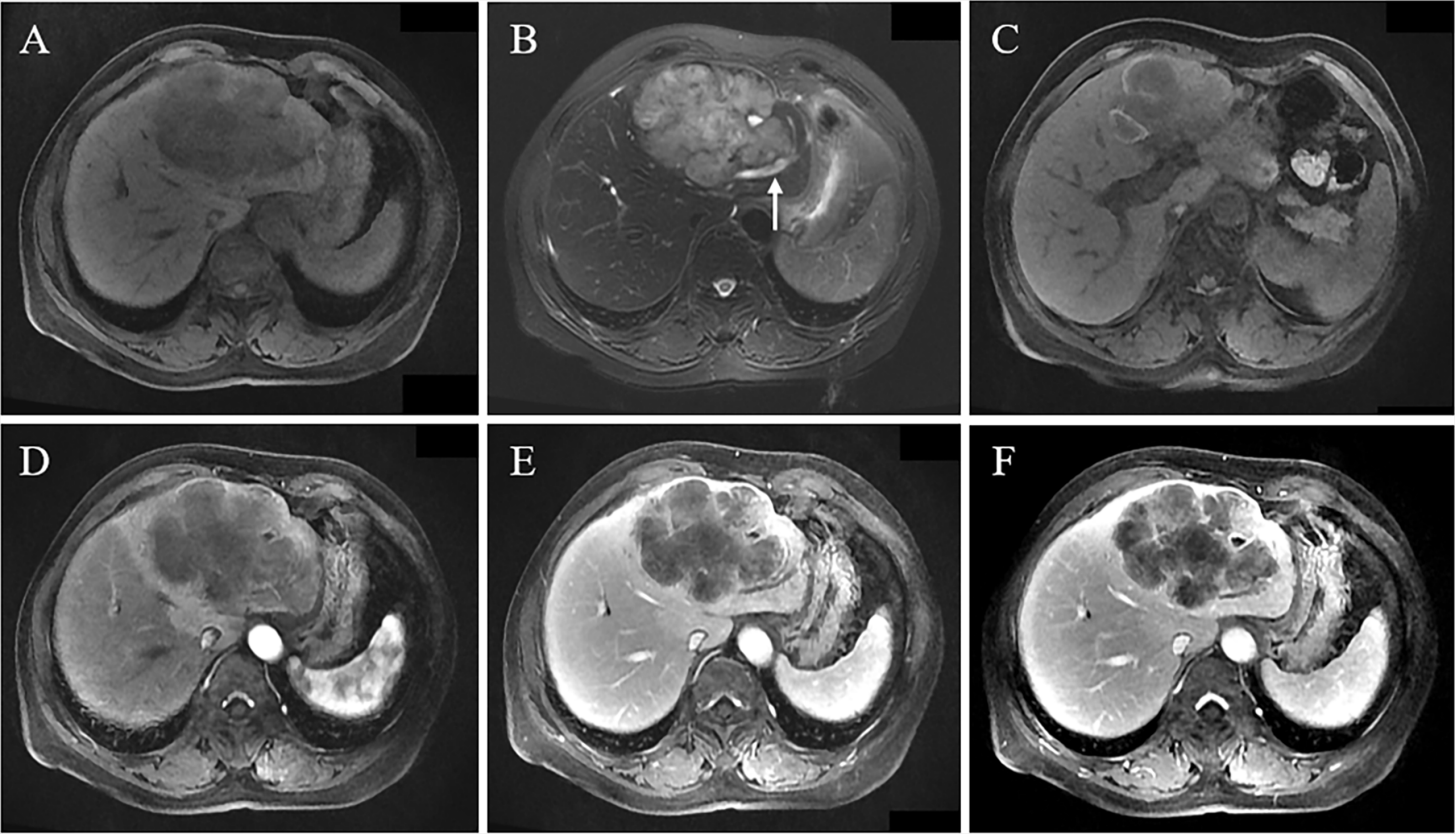
A 66-year-old female patient had abdominal pain. MRI revealed a huge lobulated mass in the left lobe, with hypointensity on T1WI **(A)**, slight hyperintensity on T2WI, and local cholangiectasis (arrow) **(B)**; local flake hyperintensity of tumor on T1WI reveals hemorrhage **(C)**; Gd-DEPA enhanced MRI scan demonstrates peripheral enhancement of tumor in arterial phase, peritumoral abnormal perfusion **(D)**, and multiple patchy enhanced regions of tumor observed in portal vein phase and delayed phase **(E, F)**, indicating the progressive enhancement pattern.

**Figure 2:**
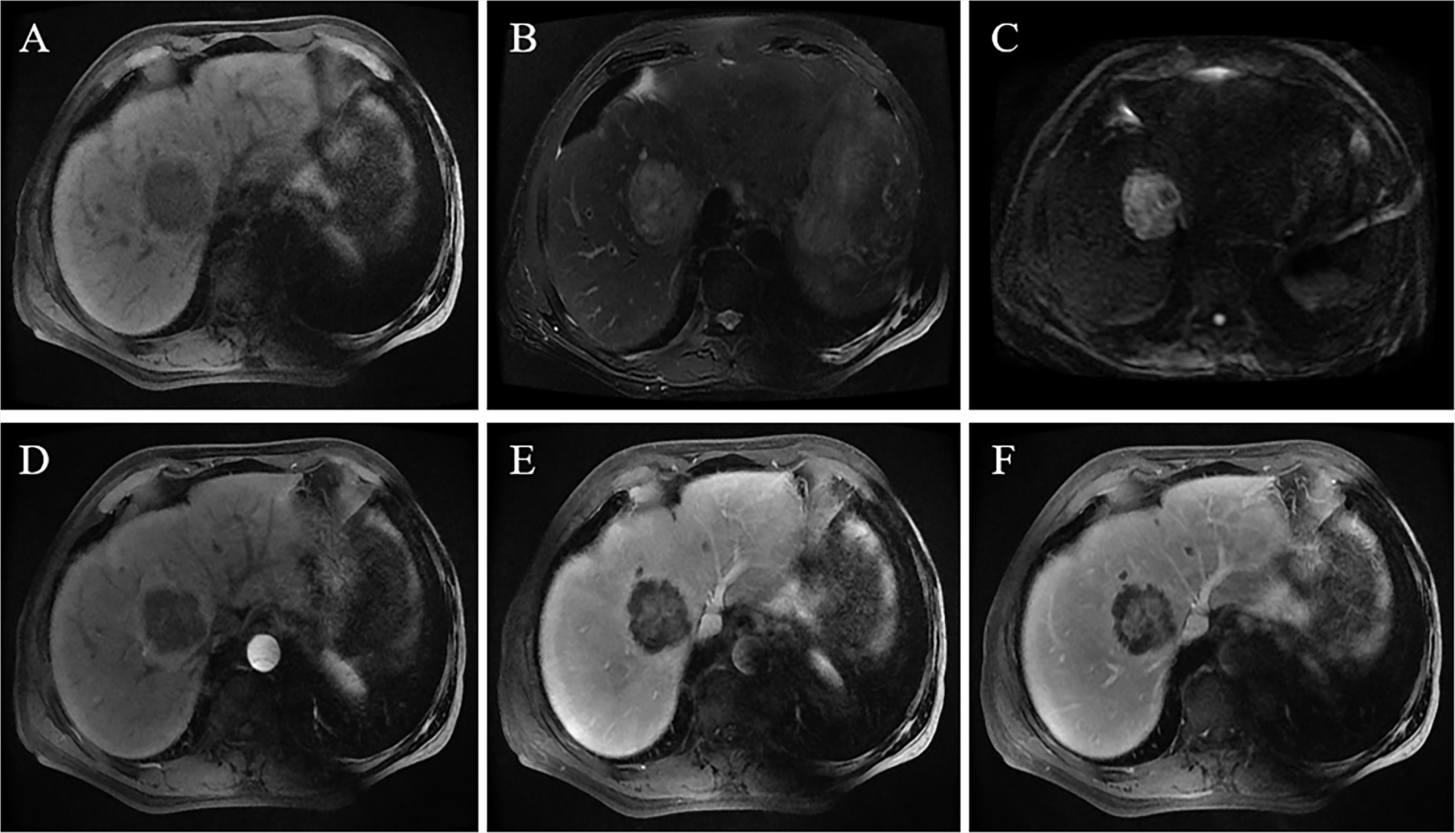
A 69-year-old male patient was accidentally found to have a liver tumor on an annual screening. An MRI scan revealed that the tumor was located in the right lobe and was 5.8 cm in size. The lesion showed a hypointense signal on T1WI **(A)**, slightly hyperintense signal on T2WI, and hepatic capsular retraction **(B)**; the tumor showed a high signal on the DWI sequence with b = 1000 s/mm² **(C)**. On contrast-enhanced MRI, the lesion shows peripheral enhancement in the arterial phase, blurred patchy hyperintense signal in the central region of the tumor **(D)**, and massive enhancement in the central region of the tumor in the portal phase and delayed phase **(E, F)**.

**Figure 3:**
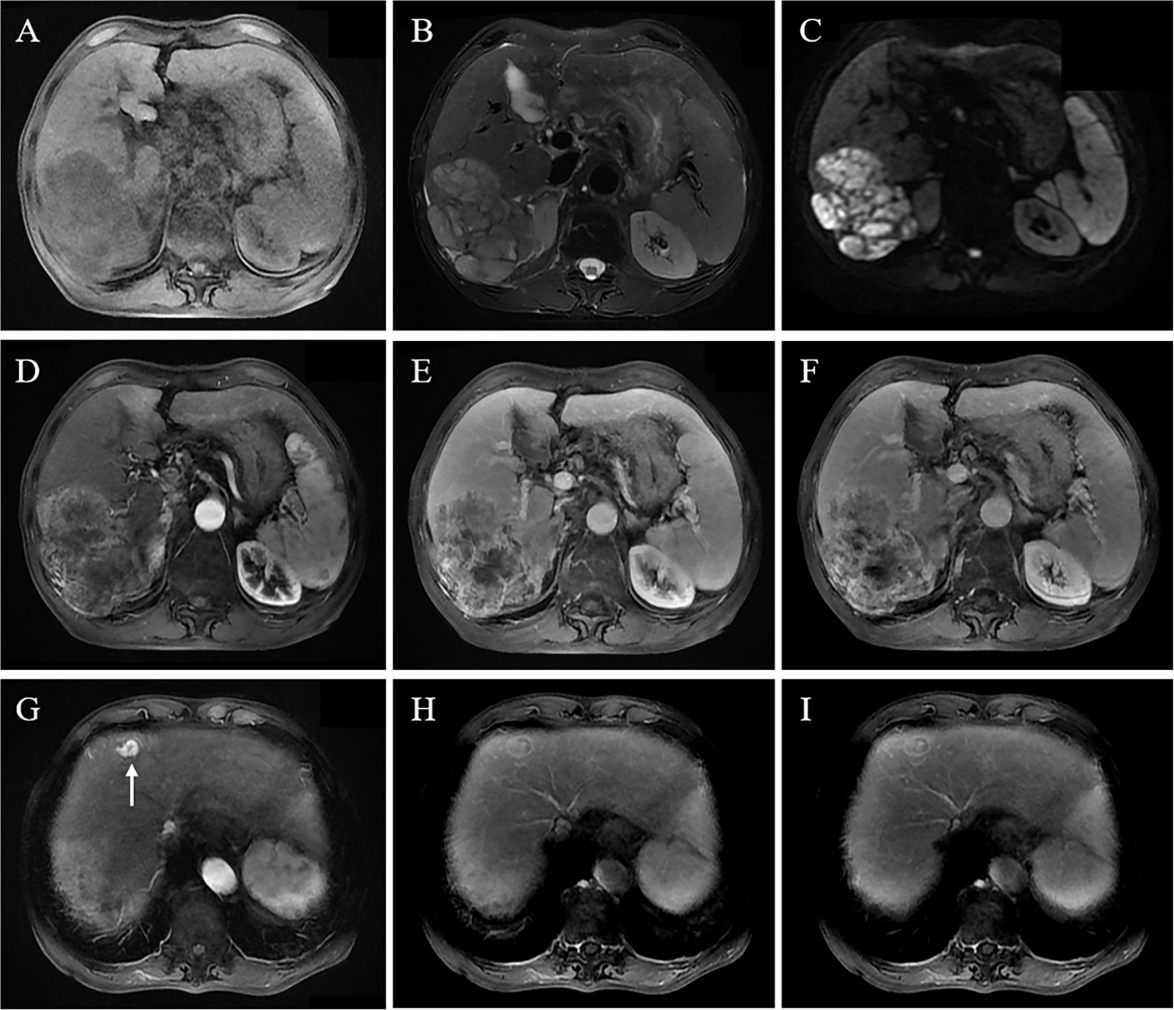
A 68-year-old male patient with chronic HBV and liver cirrhosis underwent an MRI scan because of abdominal pain. It revealed a large mass in the right lobe, 10.5 cm in size. The lesion shows a hypointense signal on T1WI **(A)**, slightly hypointense signal on T2WI **(B)**, and heterogeneously high signal on DWI sequence **(C)**; contrast-enhanced MRI shows heterogeneous enhancement in the arterial phase **(D)**; the lesion shows persistently and heterogeneously high enhancement in portal vein phase and delayed phase **(E, F)**, with persistently high enhancement pattern. Intense enhancement of a small metastatic tumor in the left liver in the arterial phase **(H)** and persistently high enhancement of a partial lesion in the portal vein phase and delayed phase with an enhanced pseudocapsule **(G, I)**.

**Figure 4:**
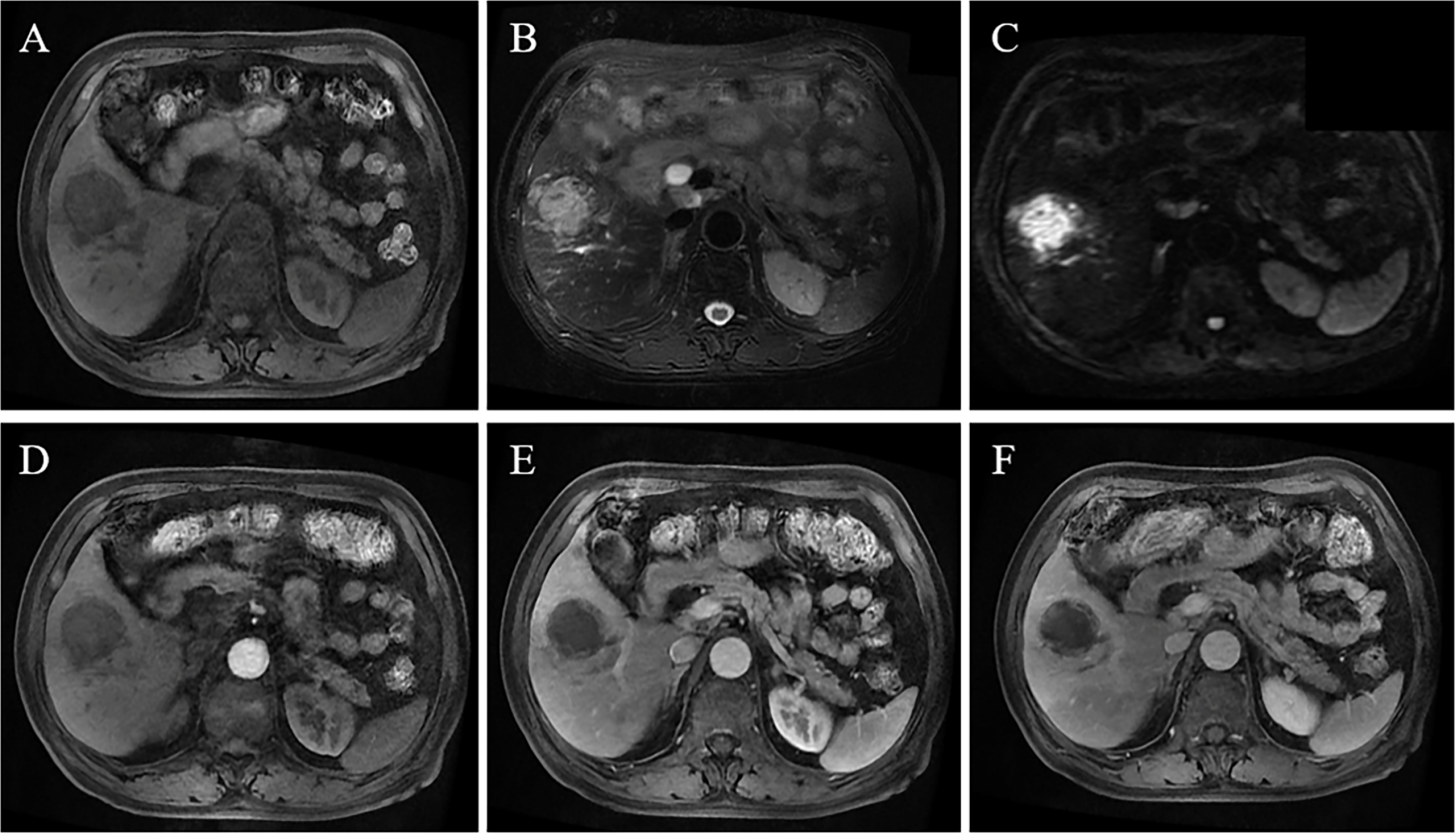
A 75-year-old male patient had fever symptoms. MRI reveals a 4.4-cm mass in the right lobe, with hypointensity on T1WI **(A)**, slightly hyperintense signal on T2WI **(B)**, and high signal on DWI **(C)**; contrast-enhanced MRI shows low enhancement in the arterial phase **(D)**, portal vein phase and delayed phase **(E, F)**, indicating a poor blood supply.

Among the 17 HSC cases, 10 showed partial enhancement in the arterial phase, 5 showed peripheral enhancement, and 2 revealed low enhancement. In addition, all 17 HSC cases showed heterogeneous enhancement in the arterial phase. Ten HSC cases showed abnormal perfusion, and eight had pseudocapsules. Five HSC cases showed vein tumor thrombi. The tumor enhancement pattern was progressive in ten cases, was persistently high in five cases, and had poor blood supply in two cases (**Figures 1**–**4**). The imaging features of the three groups are summarized in **Table 2**.

### Comparison of MRI Features

**Figure 5:**
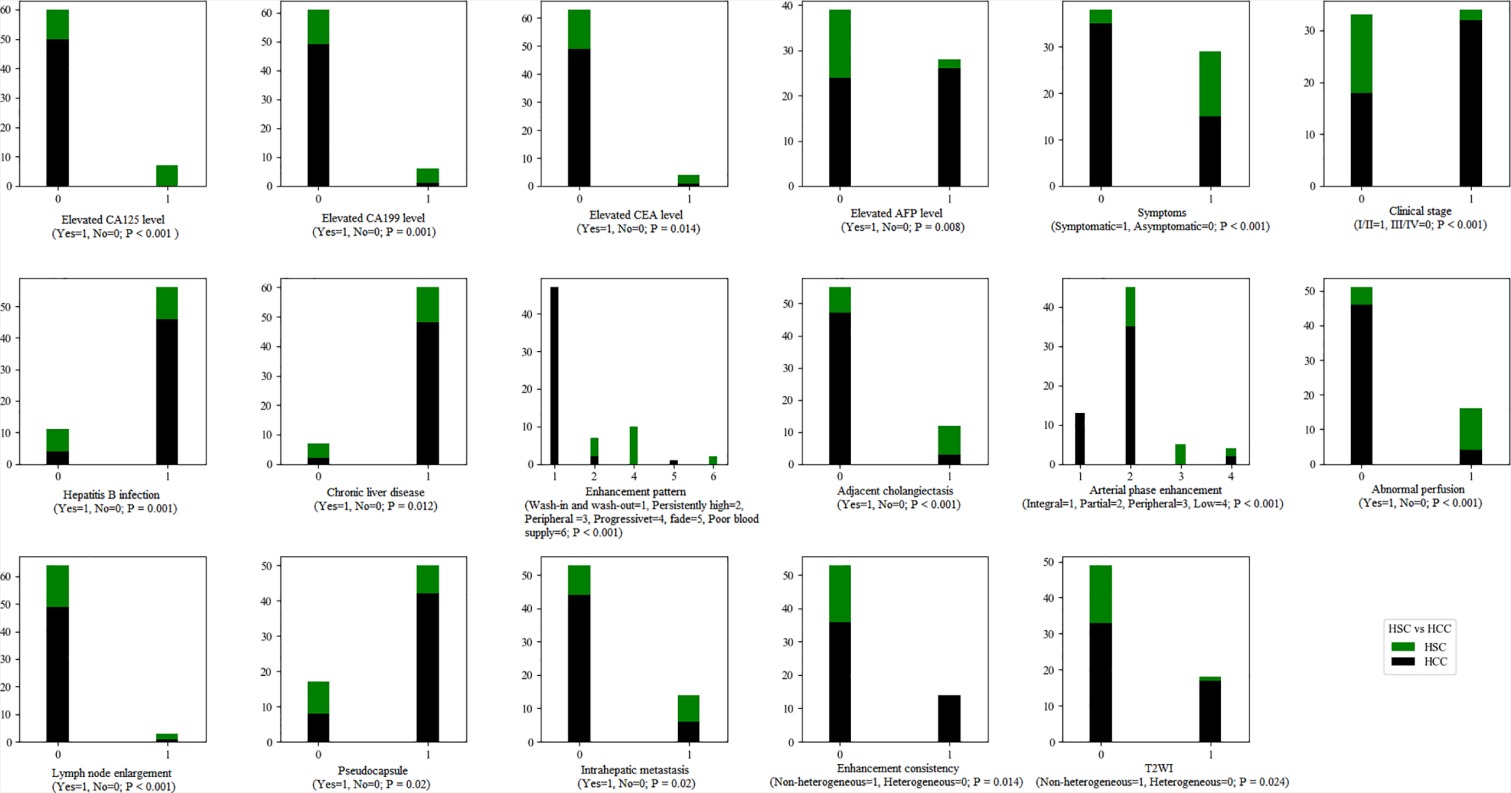
The statistical graphs showed 8 clinical features and 9 radiological features with significant differences between the HSC and ICC groups.

There were significant differences in arterial phase enhancement, enhancement pattern, abnormal perfusion, pseudocapsule, adjacent cholangiectasis, intrahepatic metastasis and lymph node enlargement between the HSC and HCC groups (*p* < 0.01), while there were significant differences in arterial phase consistency, maximum diameter and signal consistency on T2WI and in the arterial phase between the HSC and HCC groups (*p* < 0.05) (**Figure 5**).

There were no significant differences in number, tumor site, morphology, signal intensity (T1WI and DWI), target sign, tumor boundary, intratumoral fat, hemorrhage, necrosis, HCR, vein tumor thrombus or mosaic sign between the HSC and HCC groups.

**Figure 6:**
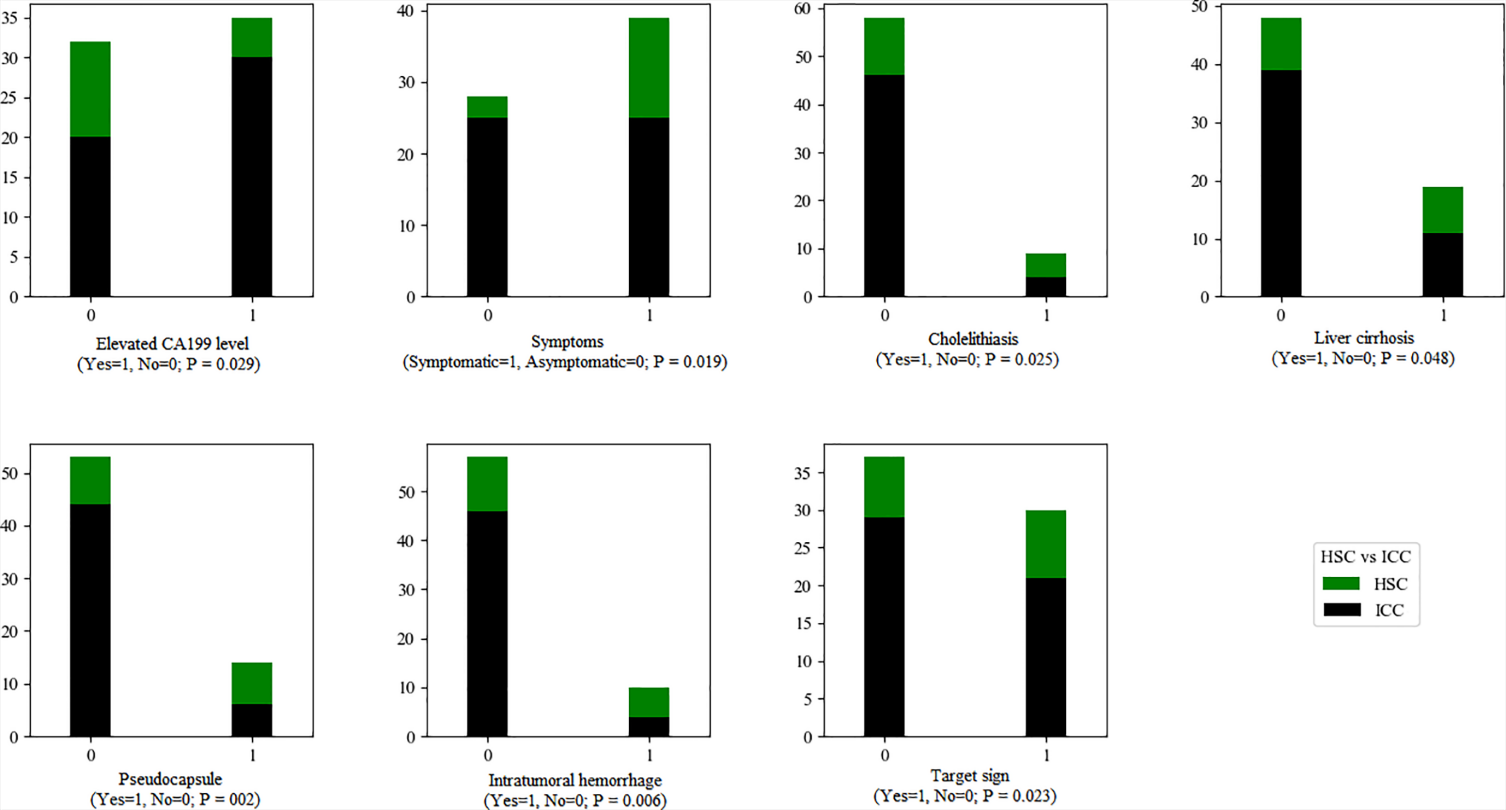
The statistical graphs showed 4 clinical features and 3 radiological features with significant differences between the HSC and ICC groups.

There were significant differences in intratumoral hemorrhage, pseudocapsule (both *p* < 0.01) and target sign (*p* < 0.05) between the HSC and ICC groups (**Figure 6**).

There were no significant differences in number, tumor site, maximum diameter, morphology, signal intensity and heterogeneity (T1WI, T2WI, DWI), tumor boundary, adjacent cholangiectasis, intratumoral necrosis, arterial phase enhancement, arterial phase enhancement consistency, abnormal perfusion, peripheral wash-in and wash-out, HCR, vein tumor thrombus, enhancement pattern, mosaic sign, intrahepatic metastasis or lymph node enlargement between the HSC and ICC groups.

## Discussion

Only approximately 10% of HSC cases suffered from abnormally elevated AFP, which was significantly lower than that in the HCC cases in our study. Our results also showed that a small proportion of HSC cases had abnormally elevated CEA and CA199 levels. In previous studies, the incidence rates of abnormal AFP among HSC cases varied greatly, between 17.5% and 40% (3, 4, 11). These results indicated that the diagnostic value of AFP for HSCs was relatively less than that for HCC. Therefore, imaging examination was important for the diagnosis of HSC cases. At the same time, a meta-analysis revealed that imaging scans combined with AFP could remarkably improve the detection rate of liver cancer compared with a single examination (12).

Previous studies have suggested that tumor differentiation and vascular invasion are closely correlated with preoperative serum AFP levels, and HCC cases with elevated serum AFP levels usually have a poor prognosis (13, 14). However, a univariate analysis enrolling 126 HSC cases did not prove the elevated serum AFP level to be an independent predictor of recurrence-free survival (4). Therefore, plasma AFP has limited diagnostic and prognostic value for HSC cases. Similar to our research, a small proportion of HSC cases showed elevated CEA and CA-199 levels, related to the cholangiocarcinoma-related subtypes of HSC (3, 7). These results suggested that CEA and CA199 had little value in the differential diagnosis of HSC and ICC cases.

Symptomatic HSC cases account for approximately 68% to 75% of cases, with abdominal pain being the most common symptom (3, 5, 10, 11). Similarly, this study confirmed that HSCs tended to show clinical symptoms. In our study, approximately half of our HSC cases had hepatitis B infection, which was apparently more frequent than that in the HCC group. However, a recent study revealed that there was no significant difference in the incidence rate of hepatitis virus infection or liver cirrhosis between HSC and HCC cases (4). Therefore, we believe that HSCs share certain tumorigenesis paths similar to those of HCC, but the pathogenic factors of HSCs are more diversified.

Our results showed that more than half of HSC cases showed progressive enhancement on Gd-DTPA-enhanced MRI scans, and approximately 1 of 3 of cases displayed persistently high enhancement. Similar to our study, a study enrolling 10 cases reported that the HSC CT/MRI enhancement pattern mainly manifested as “progressive” (15). However, in an imaging study with 24 cases, the HSC CT/MRI enhancement pattern was dominated by a “peripheral” pattern (9). The differences in the frequency of these enhancement patterns could be related to the small sample size. Furthermore, in an HSC subgroup study, sarcomatous ICC mainly manifested as gradual centripetal enhancement on contrast-enhanced CT (7). Therefore, we speculated that such progressive enhancement could help to distinguish HSCs from HCC but might be confused with ICC.

HSC was apparently larger in size than HCC in our study. The literature also verified that HSCs had a large size, with an average value of ~ 8 cm (6, 9, 10, 15, 16), except for a group of cases with a relatively small size (less than 5 cm) (8). Therefore, a larger size could be used to distinguish HSCs from HCC, but the radiological features of small HSC cases were similar to those of HCC (8). In our cases, the discovery of HCR contributed to the diagnosis of HSC, which could be related to the ICC origin of HSC. Similar to our research, Seo reported a small proportion (only 6.5%) of HCR (8). However, the diagnostic value of HCR in HSCs should be further determined by continuously increasing HSC studies.

Compared with ICC, the HSCs in this study showed a high proportion of intratumoral hemorrhage, consistent with a previous study (16). However, the incidence rate of intratumoral hemorrhage in HSCs was reportedly 21.7%, without a significant difference in a comparative study (8). We believe that this difference could be attributed to the smaller diameter of HSCs in their study (average, 4.8 cm) compared with our cases and other studies. The discovery of pseudocapsules contributed to the differential diagnosis between HSCs and ICCs, the incidence rate of which was 15.2% to 50.0% in different studies (8, 11, 15). Notably, when the HSC size was small, the incidence rate of pseudocapsules was low (8). Additionally, in our study, HSC likely led to lymph node enlargement, but the incidence rate was slightly lower than that with ICC. A clinical study further verified that the incidence rate of lymph node metastasis was as high as 25.0% (11). Hence, for suspected HSC cases, clinicians should pay attention to lymph node status.

Certain limitations should also be noted in this retrospective study. First, the sample size was relatively small due to the low morbidity of HSCs. To the best of our knowledge, this study was a comparative study that enrolled a relatively large number of HSC cases for imaging research. Second, no quantified imaging features were used since it was our preliminary study. In the future, we will consider using radiomics methods to distinguish HSCs from HCC and ICC. Finally, due to the small sample size, differences in the imaging findings were not explored based on HSC subtypes.

In brief, the valuable clues in the diagnosis of HSC were elevated tumor markers and MRI features, including progressive enhancement, heterogeneous signal and enhancement, pseudocapsule, large size, intratumoral hemorrhage, intrahepatic metastasis, and lymph node enlargement. Clinicians should pay attention to HSC imaging findings, which might be similar to those with ICC. Detailed clinical and imaging feature analysis will help to attain an accurate diagnosis.

## Data Availability Statement

The original contributions presented in the study are included in the article/supplementary material. Further inquiries can be directed to the corresponding authors.

## Ethics Statement

The studies involving human participants were reviewed and approved by Research Ethics Review Committees of The First Affiliated Hospital of Medical College of Zhejiang University and Yiwu Center Hospital. Written informed consent for participation was not required for this study in accordance with the national legislation and the institutional requirements.

## Author Contributions

WL and WX completed conception and design, development of methodology (provided animals, acquired and managed patients, provided facilities, etc.), and analysis and interpretation of data (e.g., statistical analysis, biostatistics, computational analysis). All authors were involved in writing, review, and/or revision of the manuscript and administrative, technical, or material support (i.e., reporting or organizing data, constructing databases). All authors contributed to the article and approved the submitted version.

## Conflict of Interest

The authors declare that the research was conducted in the absence of any commercial or financial relationships that could be construed as a potential conflict of interest.
